# Crystal structure of 2,2-di­chloro-1-(piperidin-1-yl)ethanone

**DOI:** 10.1107/S205698901402708X

**Published:** 2015-01-01

**Authors:** Markus Schwierz, Helmar Görls, Wolfgang Imhof

**Affiliations:** a University Koblenz-Landau, Institute for Integrated Natural Sciences, Universitätsstrasse 1, 56070 Koblenz, Germany; b Friedrich-Schiller-University Jena, Institute of Inorganic and Analytical Chemistry, Humboldtstrasse 8, 07743 Jena, Germany

**Keywords:** crystal structure, piperidine ring, ethanone, weak hydrogen bonds, inter­molecular Cl⋯O inter­actions

## Abstract

In the title compound, C_7_H_11_Cl_2_NO, the piperidine ring shows a chair conformation and the bond-angle sum at the N atom is 359.9°. The H atom of the di­chloro­methyl group is in an eclipsed conformation with respect to the carbonyl group (H—C—C=O = −5°). In the crystal, inversion dimers are linked by pairs of C—H⋯O hydrogen bonds between the di­chloro­methyl group and the carbonyl O atom, which generate *R*_2_^2^(8) loops. The dimers are linked into a ladder-like structure propagating in the [100] direction by short O⋯Cl [3.1084 (9) Å] contacts.

## Related literature   

For the synthetic procedure, see: Schank (1967 ▸). For a survey concerning weak hydrogen bonds, see: Desiraju & Steiner (1999 ▸). For a description of the nature of inter­molecular inter­actions between chlorine and oxygen, see: Lommerse *et al.* (1996 ▸). For the crystal structure of the starting compound, see: Schwierz *et al.* (2015 ▸).
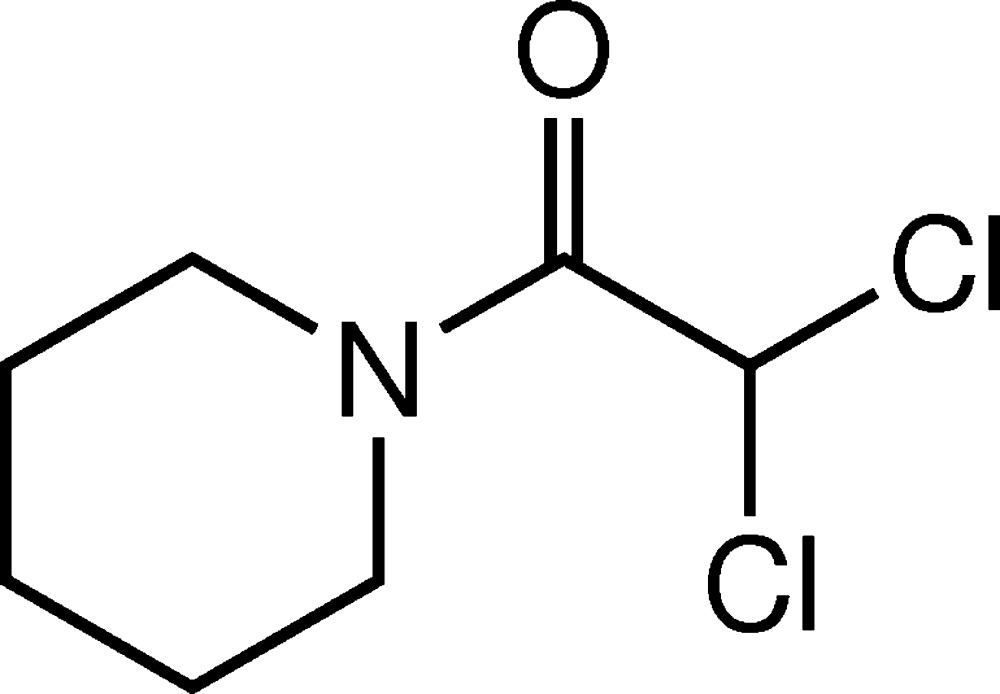


## Experimental   

### Crystal data  

C_7_H_11_Cl_2_NO
*M* *_r_* = 196.07
Monoclinic, 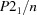

*a* = 6.2972 (1) Å
*b* = 15.4896 (2) Å
*c* = 9.3709 (2) Å
β = 108.920 (1)°
*V* = 864.66 (3) Å^3^
*Z* = 4
Mo *K*α radiation
μ = 0.69 mm^−1^
*T* = 133 K
0.08 × 0.07 × 0.06 mm

### Data collection  

Nonius KappaCCD diffractometer
Absorption correction: multi-scan (*SADABS*; Bruker, 2002 ▸) *T*_min_ = 0.712, *T*_max_ = 0.746
5528 measured reflections
1982 independent reflections
1909 reflections with *I* > 2σ(*I*)
*R*_int_ = 0.014

### Refinement  

*R*[*F* ^2^ > 2σ(*F* ^2^)] = 0.020
*wR*(*F* ^2^) = 0.051
*S* = 1.07
1982 reflections
144 parameters
All H-atom parameters refined
Δρ_max_ = 0.36 e Å^−3^
Δρ_min_ = −0.18 e Å^−3^

### 

Data collection: *COLLECT* (Nonius, 1998 ▸); cell refinement: *DENZO* (Otwinowski & Minor, 1997 ▸); data reduction: *DENZO*; program(s) used to solve structure: *SHELXS97* (Sheldrick, 2008 ▸); program(s) used to refine structure: *SHELXL97* (Sheldrick, 2008 ▸); molecular graphics: *ORTEP-3 for Windows* (Farrugia, 2012 ▸) and *Mercury* (Macrae *et al.*, 2008 ▸); software used to prepare material for publication: *SHELXL97*.

## Supplementary Material

Crystal structure: contains datablock(s) I, New_Global_Publ_Block. DOI: 10.1107/S205698901402708X/hb7336sup1.cif

Structure factors: contains datablock(s) I. DOI: 10.1107/S205698901402708X/hb7336Isup2.hkl

Supporting information file. DOI: 10.1107/S205698901402708X/hb7336Isup3.cml

**Fig. 1.**
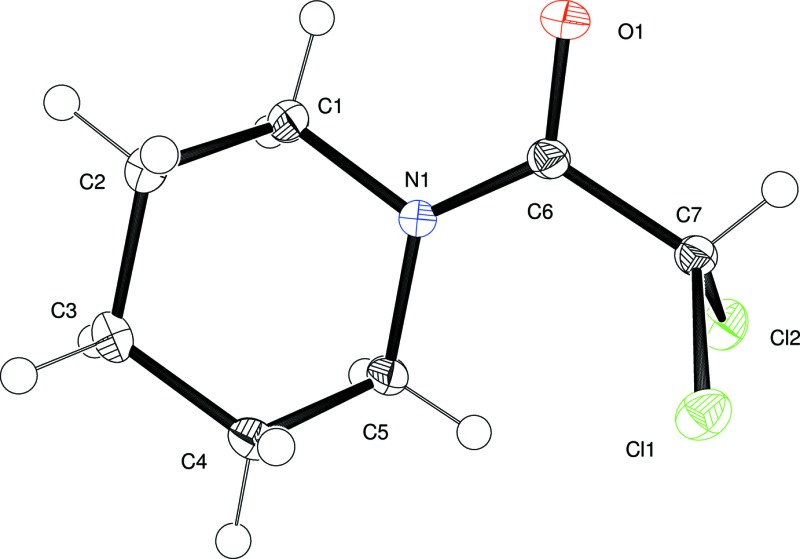
: Molecular structure of the title compound with displacement ellipsoids drawn at the 50% probability level.

**Fig. 2.**
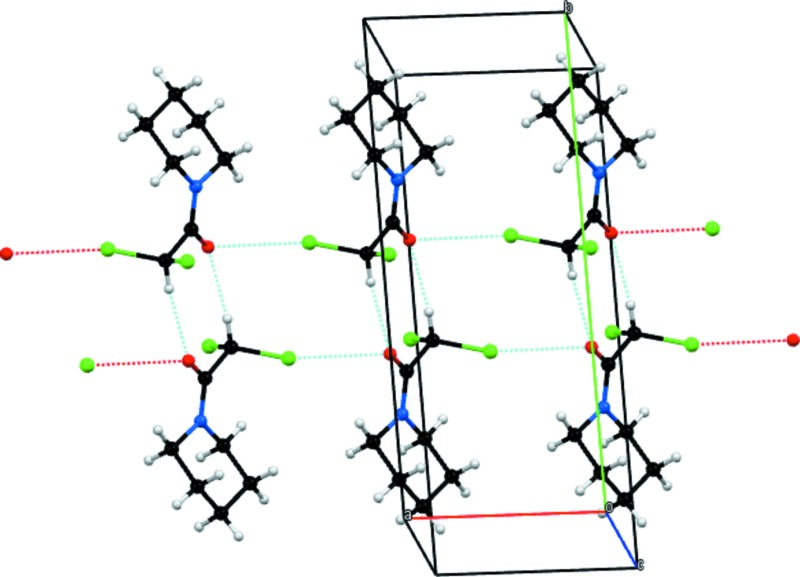
: Crystal structure of the title compound showing ladder-like arrangement parallel to the ac plane.

. DOI: 10.1107/S205698901402708X/hb7336fig1.tif
Mol­ecular structure of the title compound with displacement ellipsoids drawn at the 50% probability level.

ac . DOI: 10.1107/S205698901402708X/hb7336fig2.tif
Crystal structure of the title compound showing ladder-like arrangement parallel to the *ac* plane.

CCDC reference: 1038542

Additional supporting information:  crystallographic information; 3D view; checkCIF report

## Figures and Tables

**Table 1:** Hydrogen-bond geometry (, )

*D*H*A*	*D*H	H*A*	*D* *A*	*D*H*A*
C7H7O1^i^	0.927(13)	2.286(12)	3.1931(13)	166(1)

Symmetry code: (i) 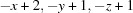.

## supplementary crystallographic information

### S1. Comment

The title compound is an intermediate in the synthesis of 2,2-dimethoxy-1-(pyridin-2-yl)ethanone and has been synthesized from 2,2-dichloro-1-(piperidin-1-yl)butane-1,3-dione (Schwierz *et al.*, 2015) following a modified procedure (Schank, 1967). As it is expected the piperidine ring shows a chair conformation and the amide substructure is planar. The hydrogen atom of the dichloromethyl group is in an eclipsed conformation with respect to the carbonyl group. In the crystal structure, dimeric aggregates are formed by hydrogen bonds of the C–H···O type between the dichloromethyl group and the carbonyl oxygen atom. In addition, these dimers are linked into a ladder-like structure parallel to the *ac* plane by oxygen chlorine contacts.

### S2. Experimental

22 ml methanol was cooled down to -6°C and then 1.93 g (84 mmol) sodium was slowly added in a way that the temperature is maintained. Afterwards 20.0 g (84 mmol) 2,2-dichloro-1-(piperidin-1-yl)butane-1,3-dione in 10 ml methanol was dropwise added to the solution of NaOMe. The resulting solution was stirred for 30 minutes and then neutralized with aqueous HCl at -10°C. After evaporating the mixture to dryness the amorphous material was collected on filter paper in a Büchner funnel and washed with water (yield: 13.6 g, 83%). The product has to be destilled *in vacuo* (0.2 mbar) and condensed into a Schlenk tube cooled by liquid nitrogen to obtain colourless prisms for X-ray diffraction.

### S3. Refinement

The positions of all hydrogen atoms have been determined from a Fourier map and all hydrogen atoms were refined without any constraints.

### Crystal data

**Table:**

C_7_H_11_Cl_2_NO	*Z* = 4
*M**_r_* = 196.07	*F*(000) = 408
Monoclinic, *P*2_1_/*n*	*D*_x_ = 1.506 Mg m^−^^3^
Hall symbol: -P 2yn	Mo *K*α radiation, λ = 0.71073 Å
*a* = 6.2972 (1) Å	µ = 0.69 mm^−^^1^
*b* = 15.4896 (2) Å	*T* = 133 K
*c* = 9.3709 (2) Å	Prism, colourless
β = 108.920 (1)°	0.08 × 0.07 × 0.06 mm
*V* = 864.66 (3) Å^3^	

### Data collection

**Table:**

Nonius KappaCCD diffractometer	1982 independent reflections
Radiation source: fine-focus sealed tube	1909 reflections with *I* > 2σ(*I*)
Graphite monochromator	*R*_int_ = 0.014
phi– + ω–scan	θ_max_ = 27.5°, θ_min_ = 2.6°
Absorption correction: multi-scan (*SADABS*; Bruker, 2002)	*h* = −8→8
*T*_min_ = 0.712, *T*_max_ = 0.746	*k* = −17→20
5528 measured reflections	*l* = −12→8

### Refinement

**Table:**

Refinement on *F*^2^	Primary atom site location: structure-invariant direct methods
Least-squares matrix: full	Secondary atom site location: difference Fourier map
*R*[*F*^2^ > 2σ(*F*^2^)] = 0.020	Hydrogen site location: difference Fourier map
*wR*(*F*^2^) = 0.051	All H-atom parameters refined
*S* = 1.07	*w* = 1/[σ^2^(*F*_o_^2^) + (0.0187*P*)^2^ + 0.3796*P*] where *P* = (*F*_o_^2^ + 2*F*_c_^2^)/3
1982 reflections	(Δ/σ)_max_ = 0.001
144 parameters	Δρ_max_ = 0.36 e Å^−^^3^
0 restraints	Δρ_min_ = −0.18 e Å^−^^3^

### Special details

**Table:**

Geometry. All e.s.d.'s (except the e.s.d. in the dihedral angle between two l.s. planes) are estimated using the full covariance matrix. The cell e.s.d.'s are taken into account individually in the estimation of e.s.d.'s in distances, angles and torsion angles; correlations between e.s.d.'s in cell parameters are only used when they are defined by crystal symmetry. An approximate (isotropic) treatment of cell e.s.d.'s is used for estimating e.s.d.'s involving l.s. planes.
Refinement. Refinement of *F*^2^ against ALL reflections. The weighted *R*-factor *wR* and goodness of fit *S* are based on *F*^2^, conventional *R*-factors *R* are based on *F*, with *F* set to zero for negative *F*^2^. The threshold expression of *F*^2^ > σ(*F*^2^) is used only for calculating *R*-factors(gt) *etc*. and is not relevant to the choice of reflections for refinement. *R*-factors based on *F*^2^ are statistically about twice as large as those based on *F*, and *R*- factors based on ALL data will be even larger.

### Fractional atomic coordinates and isotropic or equivalent isotropic displacement parameters (Å^2^)

**Table:**

	*x*	*y*	*z*	*U*_iso_*/*U*_eq_	
Cl1	0.58896 (4)	0.397515 (17)	0.60904 (3)	0.01933 (8)	
Cl2	1.00866 (4)	0.453542 (17)	0.83711 (3)	0.01976 (8)	
O1	1.06874 (14)	0.38479 (5)	0.48067 (9)	0.02086 (18)	
N1	1.05601 (15)	0.28336 (6)	0.65192 (10)	0.01611 (18)	
C1	1.20371 (19)	0.22440 (7)	0.60412 (13)	0.0191 (2)	
H1B	1.333 (2)	0.2132 (9)	0.6929 (16)	0.023 (3)*	
H1A	1.254 (2)	0.2541 (9)	0.5296 (16)	0.021 (3)*	
C2	1.0825 (2)	0.14060 (7)	0.54290 (13)	0.0187 (2)	
H2B	0.959 (2)	0.1519 (9)	0.4527 (17)	0.023 (3)*	
H2A	1.189 (2)	0.1027 (9)	0.5171 (17)	0.026 (4)*	
C3	0.99241 (19)	0.09891 (7)	0.65911 (13)	0.0185 (2)	
H3B	0.910 (2)	0.0471 (9)	0.6184 (15)	0.019 (3)*	
H3A	1.119 (2)	0.0834 (9)	0.7476 (16)	0.022 (3)*	
C4	0.84334 (18)	0.16225 (7)	0.70759 (12)	0.0170 (2)	
H4B	0.713 (2)	0.1755 (9)	0.6231 (15)	0.019 (3)*	
H4A	0.792 (2)	0.1379 (9)	0.7849 (16)	0.022 (3)*	
C5	0.96872 (19)	0.24606 (7)	0.76638 (12)	0.0167 (2)	
H5B	1.098 (2)	0.2349 (9)	0.8562 (15)	0.018 (3)*	
H5A	0.875 (2)	0.2867 (9)	0.7911 (15)	0.018 (3)*	
C6	1.00703 (17)	0.36088 (7)	0.58641 (11)	0.0140 (2)	
C7	0.87376 (17)	0.42766 (7)	0.64343 (11)	0.0141 (2)	
H7	0.874 (2)	0.4785 (8)	0.5911 (14)	0.013 (3)*	

### Atomic displacement parameters (Å^2^)

**Table:**

	*U*^11^	*U*^22^	*U*^33^	*U*^12^	*U*^13^	*U*^23^
Cl1	0.01372 (13)	0.02241 (14)	0.02175 (14)	−0.00113 (10)	0.00559 (10)	−0.00364 (10)
Cl2	0.02030 (14)	0.01973 (14)	0.01757 (13)	−0.00162 (10)	0.00382 (10)	−0.00656 (9)
O1	0.0272 (4)	0.0187 (4)	0.0225 (4)	0.0016 (3)	0.0162 (3)	0.0043 (3)
N1	0.0203 (4)	0.0119 (4)	0.0204 (4)	0.0008 (3)	0.0126 (4)	0.0009 (3)
C1	0.0189 (5)	0.0149 (5)	0.0278 (6)	0.0014 (4)	0.0136 (5)	0.0003 (4)
C2	0.0213 (5)	0.0154 (5)	0.0218 (5)	0.0021 (4)	0.0103 (4)	−0.0008 (4)
C3	0.0214 (5)	0.0125 (5)	0.0214 (5)	−0.0016 (4)	0.0065 (4)	0.0003 (4)
C4	0.0181 (5)	0.0167 (5)	0.0173 (5)	−0.0017 (4)	0.0071 (4)	0.0027 (4)
C5	0.0226 (5)	0.0142 (5)	0.0160 (5)	0.0003 (4)	0.0101 (4)	0.0020 (4)
C6	0.0137 (5)	0.0138 (5)	0.0152 (5)	−0.0025 (4)	0.0054 (4)	−0.0012 (4)
C7	0.0150 (5)	0.0136 (5)	0.0146 (5)	−0.0014 (4)	0.0057 (4)	−0.0004 (4)

### Geometric parameters (Å, º)

**Table:**

Cl1—C7	1.7786 (10)	C2—H2A	0.977 (15)
Cl2—C7	1.7823 (10)	C3—C4	1.5254 (15)
O1—C6	1.2328 (13)	C3—H3B	0.966 (14)
N1—C6	1.3383 (14)	C3—H3A	0.974 (15)
N1—C5	1.4726 (13)	C4—C5	1.5264 (15)
N1—C1	1.4731 (13)	C4—H4B	0.961 (14)
C1—C2	1.5208 (15)	C4—H4A	0.961 (14)
C1—H1B	0.973 (15)	C5—H5B	0.980 (14)
C1—H1A	0.971 (14)	C5—H5A	0.942 (14)
C2—C3	1.5249 (15)	C6—C7	1.5332 (14)
C2—H2B	0.960 (15)	C7—H7	0.927 (13)
			
C6—N1—C5	126.95 (9)	C3—C4—C5	110.99 (9)
C6—N1—C1	119.41 (9)	C3—C4—H4B	109.7 (8)
C5—N1—C1	113.57 (8)	C5—C4—H4B	108.7 (8)
N1—C1—C2	110.77 (9)	C3—C4—H4A	111.2 (8)
N1—C1—H1B	106.7 (8)	C5—C4—H4A	108.9 (8)
C2—C1—H1B	110.4 (8)	H4B—C4—H4A	107.2 (11)
N1—C1—H1A	108.1 (8)	N1—C5—C4	110.02 (9)
C2—C1—H1A	112.0 (8)	N1—C5—H5B	106.9 (8)
H1B—C1—H1A	108.8 (12)	C4—C5—H5B	110.5 (8)
C1—C2—C3	110.46 (9)	N1—C5—H5A	109.2 (8)
C1—C2—H2B	109.9 (9)	C4—C5—H5A	111.4 (8)
C3—C2—H2B	109.0 (8)	H5B—C5—H5A	108.8 (11)
C1—C2—H2A	107.8 (8)	O1—C6—N1	123.54 (10)
C3—C2—H2A	111.4 (8)	O1—C6—C7	115.35 (9)
H2B—C2—H2A	108.3 (12)	N1—C6—C7	121.09 (9)
C2—C3—C4	110.35 (9)	C6—C7—Cl1	113.17 (7)
C2—C3—H3B	110.4 (8)	C6—C7—Cl2	111.88 (7)
C4—C3—H3B	110.3 (8)	Cl1—C7—Cl2	111.30 (5)
C2—C3—H3A	108.8 (8)	C6—C7—H7	107.1 (8)
C4—C3—H3A	108.4 (8)	Cl1—C7—H7	107.6 (8)
H3B—C3—H3A	108.6 (11)	Cl2—C7—H7	105.4 (8)

### Hydrogen-bond geometry (Å, º)

**Table:**

*D*—H···*A*	*D*—H	H···*A*	*D*···*A*	*D*—H···*A*
C7—H7···O1^i^	0.927 (13)	2.286 (12)	3.1931 (13)	166 (1)

Symmetry code: (i) −*x*+2, −*y*+1, −*z*+1.
