# Supplementary figures and images for: Crystal structure of 2,2-di­chloro-1-(piperidin-1-yl)ethanone
Source: Acta Crystallogr E Crystallogr Commun. 2015 Jan 1;71(Pt 1):o47. doi: 10.1107/S205698901402708X (PMC4331879; doi:10.1107/S205698901402708X)

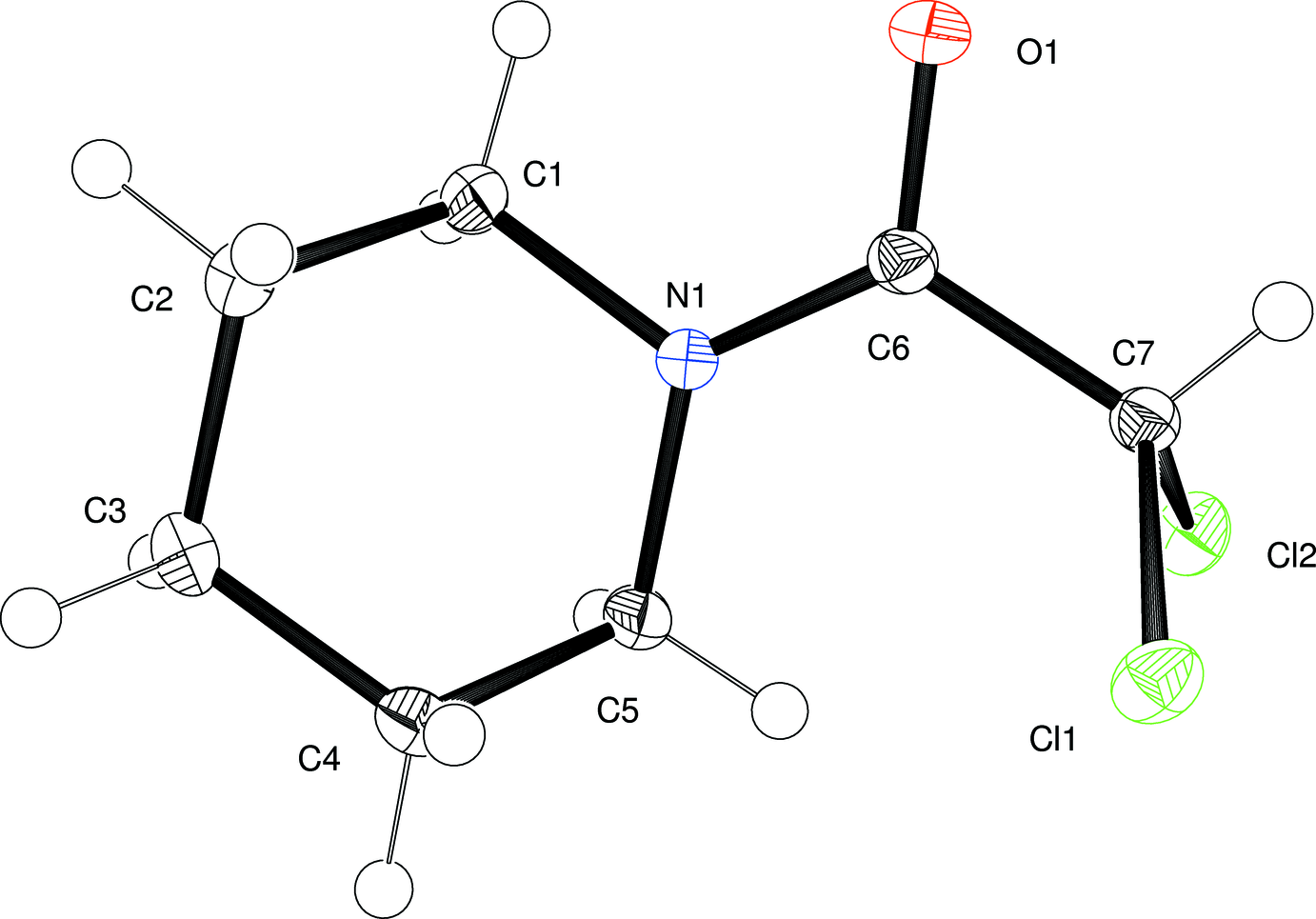

Supplement: Supplementary file 4 [file e-71-00o47-fig1.tif]

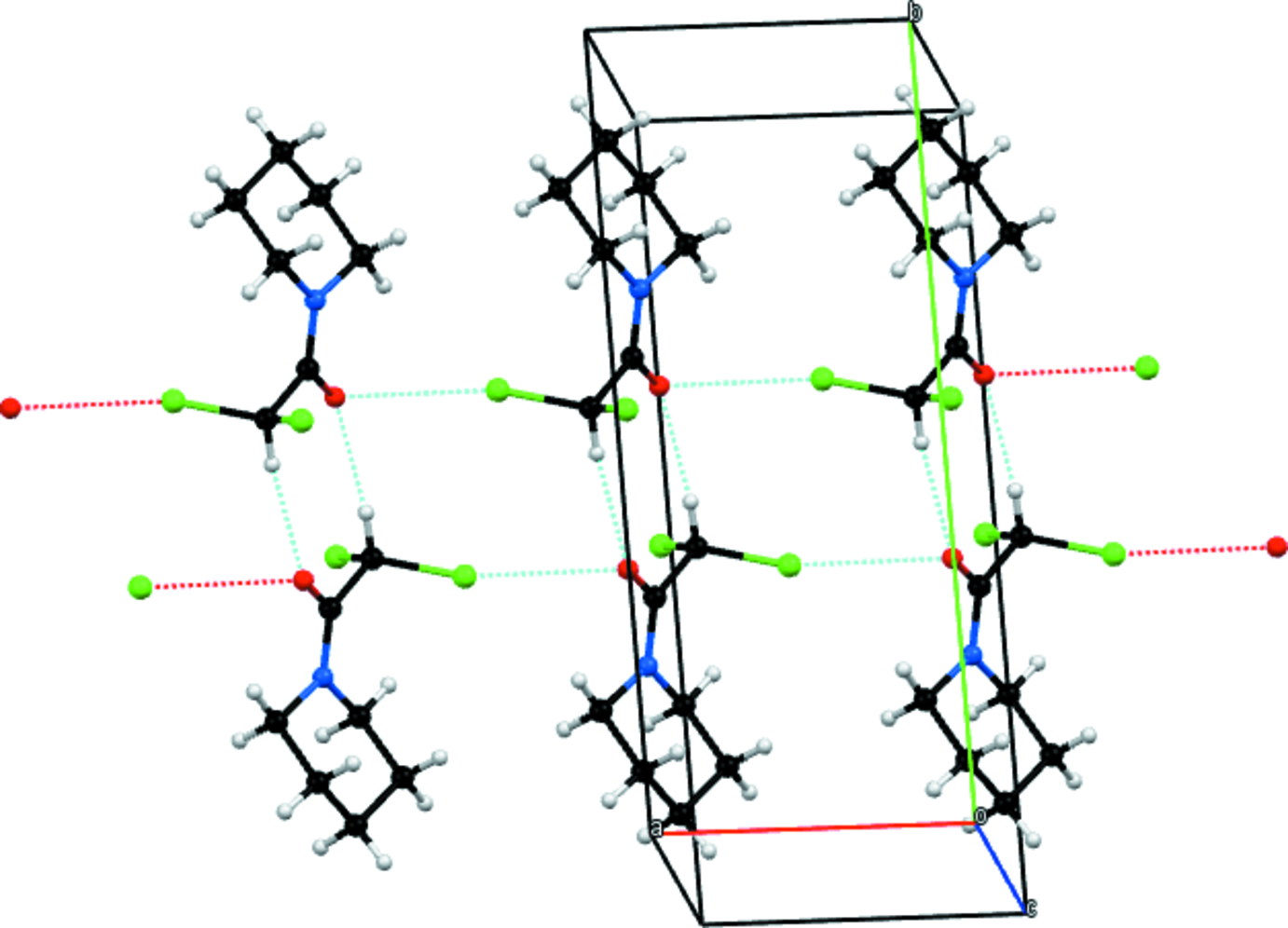

Supplement: Supplementary file 5 [file e-71-00o47-fig2.tif]
